# Assessment of vocation of rifabutin and rifapentine in replace of rifampcin in drug resistance leprosy patients: a molecular simulation study

**DOI:** 10.22099/mbrc.2017.4084

**Published:** 2017-09

**Authors:** Partha Sarathi Mohanty, Farah Naaz, Avi Kumar Bansal, Umesh Datta Gupta

**Affiliations:** 1 Microbiology and Molecular Biology National JALMA Institute for Leprosy and Other Mycobacterial Diseases Agra, India; 2 Medical Division National JALMA Institute for Leprosy and Other Mycobacterial Diseases Agra, India; 3 National JALMA Institute for Leprosy and Other Mycobacterial Diseases Agra, India

**Keywords:** Rifampicin, Rifabutin, Rifapentine, Leprosy, Drug resistance, Docking

## Abstract

The emergence of drug resistance in leprosy is a major hurdle in leprosy elimination programme. Although the problem of drug resistance is presently not acute, it is important that we collect data more systematically and monitor the trend carefully so that effective measures to combat this problem can be developed. The present study aimed at the explication of cross resistance of rifabutin and rifapentine to rifampicin which would be helpful to programme managers for implementing rifabutin or rifapentine in replace of rifampicin. In this study we built 3D model of the *M. leprae *rpoB using Swiss Model and the modelled structure was docked with rifampicin, rifabutin and rifapentine. We established that these 3 antibiotics interact with the same binding region in the modelled rpoB of *M. leprae. *Thus we conclude that vocation of rifabutin and rifapentine could not be suitable in replace of rifampicin to combat with drug resistance leprosy.

## INTRODUCTION

Current treatment for leprosy is based on standard MDT which consists of dapsone, rifampicin and clofazamine. Long term monotherapy with dapsone resulted in poor compliance, treatment failures and emergence of dapsone resistant strains of *M. leprae* [1-2]. Between the 1960’s and l970’s additional antimicrobial agents like rifampicin and clofazamine were introduced for the treatment of leprosy [3-4]. Although rifampicin proved to be a powerful anti leprosy drug by inhibiting the rpoB gene which codes for RNA polymerase β sub unit, the use of rifampicin alone or with dapsone led to the emergence of rifampicin resistance *M. leprae *strains [5]. The emergence of drug resistant put a hurdle or threat for intervention programmes implemented for infectious diseases especially in leprosy with long incubation period, social stigma and drug resistance. 

The emergence of drug resistance is a cause for concern and a threat for any infectious disease intervention programme. For leprosy, a chronic disease with social stigma, drug resistance poses a serious impediment especially at the stage where a dramatic decline in prevalence and new case detection has been achieved due to intensive and concerted chemotherapy interventions made by the national programmes and its global partners. There seems to be an extraordinary degree of complacency about drug resistance, in spite of current challenges faced by TB control programmes and the history of dapsone-resistance and its negative effects on the leprosy control strategies. This has resulted in lack of priority and absence of information on current magnitude of drug resistance in leprosy which, of course, is not evidence of an absence of drug resistance. It is assumed that a combination of three drugs, if taken regularly will prevent the emergence of drug resistance. In addition, there is limited information on patient adherence with the unsupervised components of multidrug therapy (MDT). Although the problem of drug resistance is presently not acute, it is important that we collect data more systematically and monitor the trend carefully so that effective measures to combat this problem can be developed. With the recent development of more practical and quick DNA sequencing methods to detect drug resistance, several reports of rifampicin, dapsone and ofloxacin resistance have been published which further highlights the emerging threat [2, 6-12]. Thus taking leprosy drug resistance mainly rifampicin resistance into consideration the present study aimed at the explication of cross resistance of rifabutin and rifapentine to rifampicin which would be helpful to programme managers for implementing rifabutin or rifapentine in replace of rifampicin.

## MATERIALS AND METHODS

**Figure 1:**
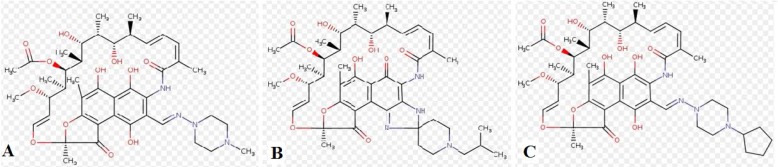
2D structure of rifampicin (A), 2D structure of rifabutin (B), 2D structure of rifapentine (C

**Data set and domain signature analysis:** The amino acid sequence of *M. leprae *rpoB (ML1891c) was retrieved from NCBI, accession no AL450380 (http://www.ncbi. nlm.nih.gov/nucleotide/). The InterProScan tool (http://www.ebi.ac.uk/Tools/pfa/ iprscan/) [13] was used to presume the protein family, super family, and domain arrangement within the protein. Conserved domains of the rpoB protein were explored by using the following databases: Pfam (http://pfam.janelia.org/)[14], SMART (http:// smart.embl-heidelberg.de/) [15], and CDD (http://www.ncbi.nlm.nih.gov/structure/cdd /cdd.shtml) [16]. Structure of rifampicin, rifabutin and rifapentine were retrieved from PDB using identifier RFP, RBT and RPT respectively (Fig. 1).

**Prediction of 3D structure: **The 3D model of the *M. leprae *rpoB was created using Swiss Model [17]. BLAST and HHBlits has been performed against the SWISS­MODEL template library for getting a perfect model from PDB identifier. For each identified template, the template's quality has been predicted from features of the target ­ template alignment. The templates with the highest quality have then been selected for model building. From the best 5 templates 4KBM was chosen for the construction of 3D model as it showed 94.40 sequence similarities with the target sequence and belongs to the rpoB of *Mycobacterium tuberculosis*. Model was built based on the target­ template alignment using ProMod3. Coordinates which are conserved between the target and the template are copied from the template to the model. Insertions and deletions are remodelled using a fragment library in the server. Side chains are then rebuilt. Finally, the geometry of the resulting model was regularized by using a force field (https://swissmodel.expasy.org/interactive).

**Model quality assessment and validation: **The quality of modelled rpoB was assessed by a number of tools to test the internal consistency and reliability of the model. ProFunc [18] analysis was performed to assess the residues fall in available zones of Ramachandran plot and also to assess the stereo chemical quality of the model and template. ERRAT tool [19] was employed to uncover the overall quality factor of the protein. Standard bond lengths and bond angles of the rpoB model were elucidated using WHAT IF (http://swift.cmbi.ru.nl/whatif/) [20] web server. ProSA tool [21] was used in the refinement and validation of the modelled structure of rpoB to check the native protein folding energy of the model by comparing the energy of the model with the potential mean force derived from a large set of known protein structures. 

**Molecular Docking: **Molecular interaction mode analysis of protein–ligand complexes is essential research in the area of structure based discovery of efficient drugs against different diseases. It is pivotal for complete comprehension of the molecular mechanisms of biological frameworks [22]. Three selected antibiotics were docked into the receptor (modelled rpoB) structure to form complex structures using MTiAutoDock server [23]. The server employs Lamarckian Genetic Algorithm (LGA) as implemented in AutoDock 4.2.6 [24] to generate orientations/conformations of compounds. The protein ligand interactions were visualized in Discovery Studio 2.5 visualizer.

## RESULTS

The rpoB of *M. leprae *retrieved from NCBI data bank was 1178 amino acids long and catalyzes the polymerisation of ribonucleotides to synthesize RNA. InterProScan search revealed that the protein is belongs to DNA directed RNA polymerase family (IPR015712). Domain predication through InterProScan and other search programmes described in the materials and methods revealed the protein is made up of 6 domains, (Ala14-Asp283). Scan programmes also elucidated rpoB recognize thioredoxine as substrate and Fe as cofactor. The Fe binding sites are 73, 104, 107, 111, 164, 198 and 201 positions of amino acid stretch. 

**Figure 2:**
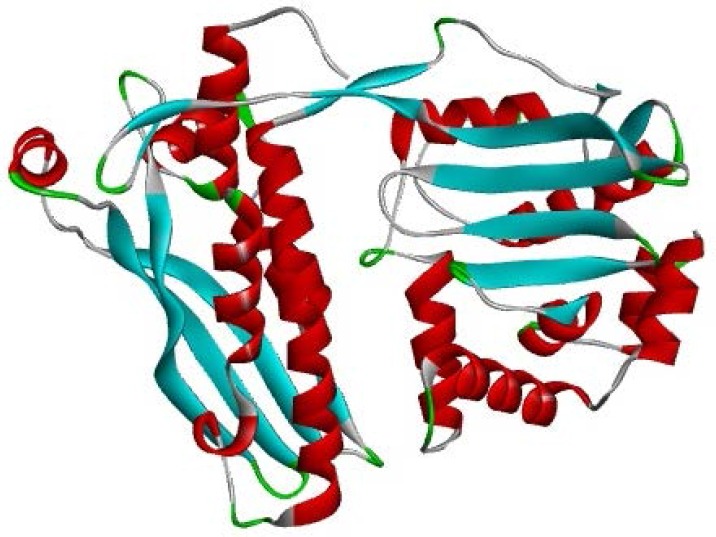
Predicted model of *M. leprae* rpoB, where helical regions represent the alpha helices and wire-like regions represent the loops

**Figure 3:**
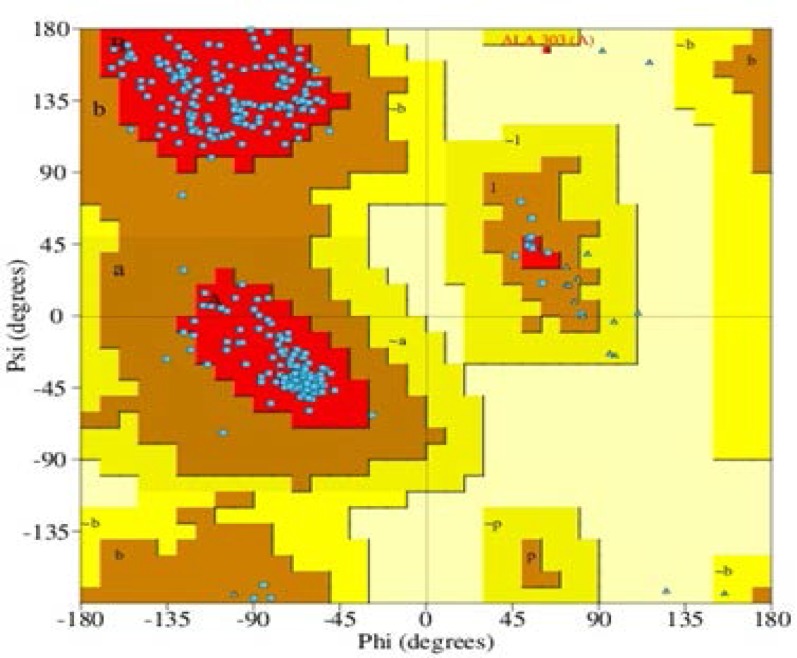
Ramachandran plot of predicted 3D structure of *M. leprae* rpoB showing the amino acid plots in most favoured regions, additional allowed region and generously allowed regions

Comparative homology modelling of protein is considered as one of the most accurate methods for 3D structure prediction, yielding suitable models for a wide spectrum of applications (Dehury et al 2013). The 3D structure of *M. leprae *rpoB was predicted from 37986 amino acid residues (Fig. 2). To validate the 3D structure Ramachandran plot was explicated (Fig. 3). The ϕ and φ angles of 319 (94.4%) residues were included in most favoured regions (A=Core alpha, B=Core beta and L= Core left-handed alpha regions), 16 (4.7%) were plotted in additional allowed region (a=Allowed alpha, b=Allowed beta, l=Allowed left-handed alpha and p=Allowed epsilon). In the generously allowed regions (~a=Generous alpha, ~b=Generous beta, ~l=Generous left-handed alpha, ~p=Generous epsilon region) 2 (0.6%) and 1 (0.3%) of amino acids fallen in the disallowed region of the plot. Comparing with the template, the built 3-D model had a similar Ramachandran plot (Table 1). The average score for dihedral angles was found to be -0.08 and main chain covalent force was 0.14. In the predicted model a total of 14 α-helices were found. Besides 14 α-helices, 5 sheets, 9 beta hairpins, 5 beta bulges, 15 strands, 27 beta turns, 1 gamma turn and 17 helix-helix interactions were found in the predicted structure.

**Table 1:** Comparisons of Ramachandran plot statistics of rpoB with its template 4KBM

**Ramachandran plot statistics**	**X-ray crystallographic structure of template 4KBM**		**Modelled rpoB of ** ***M. leprae***	
	Residues	Percentage	Residues	Percentage
Residues in most favored regions	320	94.1	319	94.4
Residues in additionally allowed regions	19	5.6	16	4.7
Residues in generously allowed regions	0	0	2	0.6
Residues in generously disallowed regions	1	0.3	1	0.3
Number of non-glycine and non-proline	340	100	338	100
Number of end residues (excluding Gly and Pro)	1		2	
Number of glycine residues	27		27	
Number of proline residues	18		18	
Overall G factor	0.23		0.06	

**Figure 4:**
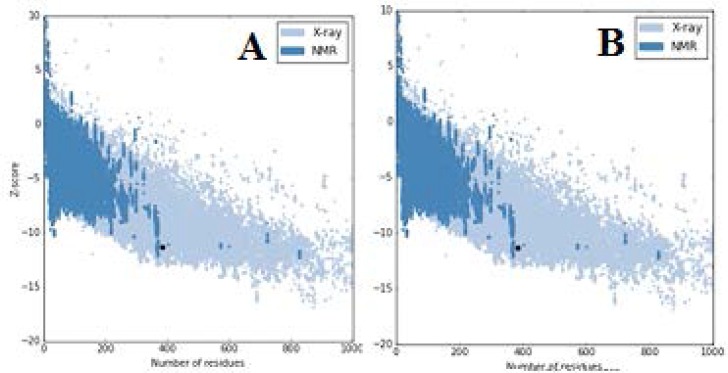
Protein Structure Analysis (ProSA) of modelled rpoB (A) Overall quality of 4KBM showing a z-score of -10.56 (Native conformation to its template). (B) Overall quality of template (4KBM) model showing a z-score of -11.32

The ERRAT score for modelled rpoB was found to be 91.112% while the reference PDB structure has a score of 93.103%. Coarse packing quality, anomalous bond length, planarity, packing quality, and the collision with symmetry axis, distribution of omega angles, proline puckering, and anomalous bond angles of the model protein were elucidated using WHAT IF server showed that the modelled rpoB is of good quality. The ProSA analysis of the modelled rpoB and template 4KBM were -10.56 and -11.23 respectively (Fig. 4).

Molecular interaction analysis of protein–ligand complexes is an essential research in the area of structure based drug discovery which provides assessment of different drug molecules to screen a lead molecule for drug of choice. Three selected drug molecules viz., rifampicin, rifabutin and rifapentine those are used in leprosy cure were asses through molecular interaction study to assess the cross resistance of rifampicin to other to compounds. The detailed of the outcomes are given bellow separately.

**Figure 5:**
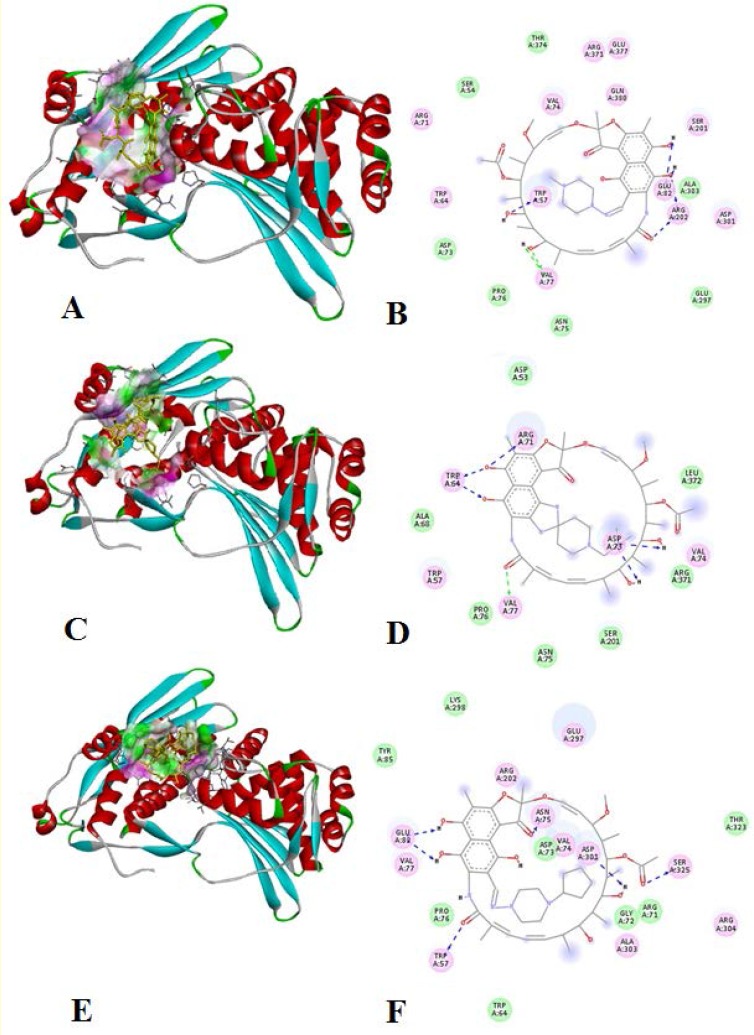
Molecular interaction of modelled rpoB with drug molecules (A, B: with rifampicin, C, D: with rifabutin, E, F: with rifapentine

The interaction analysis of modelled rpoB and rifamipicin yielded 10 active torsions and the binding energy was -12.19. Interaction study elucidated 8 electrostatic interactions (Trp-57, Trp-64, Val-77, Glu-82, Ser-201, Arg-202, Asp-301, Arg-371 and Glu-377) and 5 van der waals interactions viz., Asp-73, Val-74, Asn-75, Pro-76, Thr-374. Except these two types of interactions 10 covalent bonds were also recorded. The possible interactions of residues like Trp-57, Val-74, Ser-201, Asp-301 and Glu-377 with water molecules were also elucidated (Fig. 5A, B).

The interaction analysis of modelled rpoB and rifabutin yielded 8 active torsions and the binding energy was -8.01. Interaction study elucidated 5 electrostatic interactions (Trp-57, Trp-64, Arg-71, Asp-73 and Val-77) and 6 van der waals interactions (Val-74, Asn-75, Pro-76, Ser-201, Arg-371 and Leu-372). Except these two types of interactions 19 covalent bonds were also recorded. The possible interactions of residues like Trp-57, Arg-71 and Val-74 with water molecules were also elucidated. 

The interaction analysis of modelled rpoB and rifapentine yielded 11 active torsions and the binding energy was -11.96, Interaction study elucidated 10 electrostatic interactions (Trp-57, Val-74, Asn-75, Val-77, Glu-82, Arg-202, Glu-297, Asp-301, Arg-304 and Ser-325) and 6 van der waals interactions (Trp-64, Arg-71, Gly-72, Asp-73, Ala-303 and Thr-323). Except these two types of interactions 11 covalent bonds were also recorded. The possible interactions of residues like Asp-73, Val-74, Asn-75, Glu-297 and Asp-301 with water molecules were also elucidated.

## DISCUSSION

Leprosy is not only a curable disease, but is on the decline the world over, having eliminated from many countries. This has been possible due to the availability and wide application of MDT in 1982. Leprosy control programme has been truly a success story worldwide but the last stone is yet unturned. Leprosy cases were reported by 138 countries from all WHO regions in 2015. South-EastAsia was the highest contributor with 74% of the reported cases, followed by the Americas (14%), Africa (9%), Western Pacific (2%), and Eastern Mediterranean (1%). Moreover, the number of new cases reduced only marginally in South-East Asia between 2006 (174,118) and 2014 (154,834). India reported the highest number of new cases in 2014 (125,785; 62%of the global burden) followed by Brazil (31 064) and Indonesia (17 025) [25].

Dapsone was the only chemotherapeutic agent used for treatment of leprosy for about three decades since 1940 [26]. Clofazimine was introduced to treat leprosy in 1962 [3]. Introduction of rifampicin--a powerful bactericidal drug in 1970 has opened the avenues of multidrug therapy to treat leprosy [26]. Multidrug therapy for leprosy treatment was introduced by WHO in 1982 to combat the disease properly. Use of MDT in leprosy control programme brought down the prevalence of the disease >1.0 per 10000 population Worldwide. 

Prolonged, interrupted and inadequate use of dapsone, clofazimine and rifampicin monotherapy, leads to development of resistant strains of *M. leprae *to these drugs. The first clinically dapsone and rifampicin resistance cases were reported in 1953 [27] and 1976 [28] respectively. 

The rate of reduction in leprosy cases has slow down over the years and put a hurdle in total elimination of the disease in some countries due to non adherence of drugs and drug resistance due to mutation [29-30]. Apart from new case detection several MB patients showed drug resistant or non-respondents’ to standard MDT. Some of the non-responders had mono resistance to rifampicin or to ofloxacin or to dapsone while some patients were multi drug resistant. Although drug resistance among new cases appears to be rare, reports of single and multidrug-resistant *M. leprae* among relapse patients continue to appear in the literature [1, 31-34]. The global prevalence of drug resistance leprosy is rising slowly and after reaching highest magnitude it will create problem like MDR and XDR tuberculosis. Thus the need of the hour is to target new genes with a new and drug for the treatment of leprosy. Two other drugs viz., rifabutin and rifapentine were also used for the treatment of leprosy either alone or in combination with dapsone and clofazimine [35-39]. Thus this study aimed to establish cross resistance of rifabutin and rifapentine to rifampicin for better advocacy of these two drugs in place of rifampicin in drug resistance leprosy. The molecular interaction study conducted showed that these 3 antibiotics interact with the same binding region in the modelled rpoB of *M. leprae*. All the 3 antibiotics had an immense affinity towards the binding site observed in the interaction study as the binding energy for each of the antibiotic towards the modelled rpoB were higher. Further these three antibiotics are of same group of compounds and having minor differences in their structure. The study suggested the cross resistance of rifabutin and refapentine to rifampicin. Our results also corroborates with results of other workers [35]. Some of the researchers advocated the use of rifabutin and rifapentine in place of rifampicin in multi drug therapy in special cases like co-infection with HIV [40]. Thus we conclude that rifabutine and rifapentine could not be used in drug resistance leprosy especially in case of rifampicin resistance.
